# Digital Methodology to implement the ECOUTER engagement process

**DOI:** 10.12688/f1000research.8786.2

**Published:** 2017-01-13

**Authors:** Rebecca C. Wilson, Oliver W. Butters, Tom Clark, Joel Minion, Andrew Turner, Madeleine J. Murtagh

**Affiliations:** 1 Data 2 Knowledge Research Group, School of Social and Community Medicine, University of Bristol, Bristol, BS8 2BN, UK; 2 MRC Integrative Epidemiology Unit, University of Bristol, Bristol, BS8 2BN, UK

**Keywords:** digital methodology, Stackeholder engagement, freeware software

## Abstract

ECOUTER ( **E**mploying **CO**ncept **u**al schema for policy and **T**ranslation **E** in **R**esearch – French for ‘to listen’ – is a new stakeholder engagement method incorporating existing evidence to help participants draw upon their own knowledge of cognate issues and interact on a topic of shared concern. The results of an ECOUTER can form the basis of recommendations for research, governance, practice and/or policy. This paper describes the development of a digital methodology for the ECOUTER engagement process based on currently available mind mapping freeware software. The implementation of an ECOUTER process tailored to applications within health studies are outlined for both online and face-to-face scenarios. Limitations of the present digital methodology are discussed, highlighting the requirement of a purpose built software for ECOUTER research purposes.

## Introduction

Engaging stakeholders is understood to be essential to produce responsible practice in research as well as in business and public provision of social and health services. Stakeholder engagement brings together individuals or groups who have an interest, a stake, in a topic or issue. And it makes sense that the people most involved or most affected by research, business or public actions would best understand how these practices affect them. However, achieving effective stakeholder engagement – engagement which represents all stakeholders equally, not just the articulate and powerful, and which engages at a depth and breadth that is appropriate to the issue at hand – is known to be potentially difficult, time consuming and expensive ^1^. Most existing methods rely on being able to bring people together in real time in a single or small number of locations. Because of these difficulties, stakeholder engagement often represents only a partial understanding of an issue and may not take account of potentially important perspectives. Even when there is a genuine commitment to giving voice to diverse perspectives, stakeholder engagement may exclude the very people it seeks to involve because its structures aren’t sufficiently agile, inclusive or accessible. Our aim was to develop a method and mechanism that was simple and accessible, yet allowed for a depth of analysis needed to uncover and disentangle the complexities and nuances that emerge when bringing together numerous personal understandings and experiences to understand an issue or topic.

Employing COnceptUal schema for policy and Translation Engagement in Research (ECOUTER, http://www.bristol.ac.uk/ecouter) is a new methodology for stakeholder engagement, utilising concept and mind mapping to capture relational and associational information ^2^ to collaboratively address a question of interest in a defined stakeholder community (the method is summarised in the ECOUTER introductory video). Taken from the French verb ‘to listen’, ECOUTER brings together the knowledge, skills and experience of stakeholder contributors and supports a two-way process of informing and generating evidence and understandings of the issue in question from those who know it best. Social science methods of analysis (such as those described by Glaser ^3^) of contributions made during the engagement process are applied iteratively resulting in qualitative findings and recommended actions. The ECOUTER process as outlined below can lead to the development of recommendations for research, governance, policy and practice.

The aim of this paper is to describe the development of the digital methodology for ECOUTER, sharing the instructions for its implementation to facilitate use by others. A forthcoming paper ^4^, fully describes the research rationale underpinning the development of the ECOUTER stakeholder engagement process for digital and non-digital implementations, and includes the analysis of a number of use cases.

### The ECOUTER process

In practice ECOUTER is a four stage process based on:

1. **Engagement and knowledge exchange:** This stage involves defining a central question/issue and relevant stakeholder group(s) to facilitate discussion and contributions. The exchange in question may be undertaken online or face-to-face, though the online mechanism is anticipated to be of greatest utility for engaging stakeholders who are geographically distributed. We therefore describe the essential components for online engagement below.
2. **Analysis:** Once the ECOUTER has been conducted, the data are analysed using social science methods as described in a paper currently in press that examines the theoretical and methodological underpinnings of ECOUTER ^4^.
3. **Concept and recommendation development:** Analytic findings are then summarised in a conceptual schema; that is, a map of key concepts, their nature and relationships.
4. **Feedback and refinement:** The conceptual schema is fed back to the contributors and wider community along with recommendations for research, governance, policy and practice.

## ECOUTER technical development and implementation

### Software specification and selection

An essential aspect of the ECOUTER methodology has been the requirement for contributions to be linked/threaded within a structured discussion space. A hybrid mind-mapping-concept mapping approach offered an appropriate solution, providing a mechanism for the relationships between comments as well as enabling the comments themselves to be captured and visualised. The nature of the ECOUTER methodology necessitated the capacity to run within a number of stakeholder groups in multiple localities simultaneously. A synchronised, digital, software solution (rather than a paper-based one) was needed. A range of open-source and proprietary software exists for mind mapping. Given the importance of removing cost as a barrier to participation in an ECOUTER, we assessed and trialed a selection of open-source software and freeware solutions based on the research and user requirements outlined below. An online web-based solution rather than an installed computer program was identified as more inclusive, enabling real-time contributions across different platforms (across Windows, Linux, Mac) and from internet-enabled devices (e.g. smartphones, tablets, computers, etc.) regardless of a contributor’s physical location.

ECOUTER initiators required a simple user interface to administer, setup and manage the collaborative mind map. In addition, the data needed to be exported from the software in both image-based and text-based formats prior to analysis, with a mechanism to trace how the collaborative discussion space evolved. From a user perspective, it was essential for the software to have a simple user interface that enabled multi-user contributions within a mind map whilst retaining the anonymity of individual contributors.

The web-based collaborative mind mapping freeware Mind42 was identified as an appropriate solution to be used within the ECOUTER framework. It has a simple user interface via a website, allowing researchers to initiate an ECOUTER mind map and to manage invited contributors. Multi-user, collaborative mind maps are possible in Mind42 and the software includes versioning and a periodic history, documenting how the mind map evolves. User registration to use Mind42 requires just an email address and password to create an account, each account is assigned a unique ID comprising random letters and numbers. Users receive an email request to join an ECOUTER map. Should they choose to contribute to the mind map, the users remain anonymous both to other contributors and to the ECOUTER facilitators. Researchers administering the map only have access to the randomly generated unique ID associated with text contributions. Map facilitators are unable to identify which invited participants to the ECOUTER have registered, or made contributions, as they are unable to link the unique IDs back to individual user email addresses.

Mind42 has both a manual web accessible mechanism and an application program interface (API) to export the mind map in multiple formats. These include exports as an image, pdf, text record of all contributions, and formats compatible with other mind mapping software. Furthermore, the Mind42 native data format (a Mind42 .m42 file) is nested and hierarchical following a JSON file format, retaining metadata about the mind map including anonymised identifiers for individual contributors linked to their contributions, the number of individual contributions, and the date and time of individual contributions.

### Implementation

The Mind42 software was capable of being implemented in both an online ECOUTER and a face-to-face ECOUTER as summarised below. Full documentation on the ECOUTER wiki provides complete instructions on how to set up, run and manage an ECOUTER in either format.

***Implementation Online.*** An online ECOUTER implementation has been developed to enable running an ECOUTER over a longer time period (e.g. weeks to months or longer). It has the benefits of allowing people to contribute to discussions regardless of time zone or geographic location, and supports contributors dropping in and out of discussions over the entire time period.

**Figure 1.**
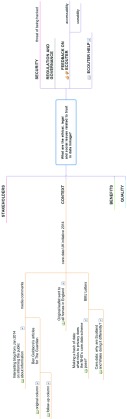
An example of a seeded ECOUTER mind map.

An ECOUTER administrator account is set up on Mind42 allowing ECOUTER facilitators to initiate, manage and moderate a mind map from start to finish. ECOUTER facilitators first seed the mind map with themes and existing evidence using the administrator account on Mind42. This task can include linking to online material including videos, photos, papers, articles and other mind maps. The seeding of the mindmap does not aim to be unbiased or representative of the debate on a topic. The purpose of seeding the mindmap is to provide participants with different entry points to the debate, but it does not try to provide a comprehensive review. In fact, the seeded evidence could be selected to be deliberately provocative in order to foster engagement. Alternately, an ECOUTER run in conjunction with a specific project or workshop will contain seeded evidence likely to have been selected accordingly and with which participants may already be somewhat familiar. Figure 1 contains an example of a seeded ECOUTER mind map based on the ECOUTER question *What are the ethical, legal and social issues related to trust in data linkage* undertaken in a pilot conducted with the Public and Population Project in Genomics and Society, (P ^3^G) in late 2014. The ECOUTER question is in a blue box in the map centre, with seeded themes in capitalised text forming the primary branches and subsequent branches containing further seeded comments and evidence in the form of web links.

Mind42 stores versions of the visual mind map periodically. At this stage, back-ups of the mind map in the desired file formats (typically as a .png and .m42 (JSON) file formats) can be taken by the ECOUTER facilitators manually after seeding the map, and regularly during the ECOUTER to prevent data loss. This is particularly important as Mind42 has no advanced user management facility and contributors are able to over-write and delete contributions made by others. Alternatively, auto-backup of the ECOUTER mind map data can be implemented at this stage using the Mind42 API, including a snapshot of the initial seeded mind map. An example back-up script is available on the ECOUTER Github repository.

Once a stakeholder group has been defined, invitations to contribute to the ECOUTER are sent by email to potential participants, facilitators additionally authorise the participant email address to access the collaborative mind map in Mind42. Should stakeholders wish to participate they would create their individual account on Mind42 using the email address used to receive their ECOUTER invitation and will automatically be granted access to the ECOUTER map.

During an ECOUTER facilitators are required to moderate the map to remove potentially disclosive or inappropriate content and edit the map structure for easier viewing e.g. colour coding branches, line wrapping long text. They also check the data backups periodically whilst the ECOUTER is running. Once the ECOUTER period is finished, facilitators close the mind map to new contributions and check the final data backups. An open source script available on the ECOUTER Github repository is used to flatten the native Mind42 data file, creating a human-readable table (.csv) of the mind map metadata, individual text contributions and preserving the final map structure. The data are then imported into computer assisted qualitative research tools (e.g. NVivo) for analysis, with a second copy archived.

***Implementation face to face.*** A face-to-face implementation has been developed to run an ECOUTER over a shorter time period from hours to one day. It has the benefits of allowing people to contribute to discussions within an exhibition-style setting, which may be placed in a high traffic public place, conference or exhibition venue.

ECOUTER facilitators initiate, manage and arrange data backups in the same manner as outlined previously. Facilitators create a number of generic ECOUTER participant accounts on Mind42 through which individuals can contribute to the mind map. Internet enabled laptops and/or tablets, provided as part of an ECOUTER exhibition stand are each logged into the ECOUTER using these accounts on Mind42, thus allowing anonymous contributions to the mind map.

In an exhibition setting it is also possible to publish the mind map online on the Mind42 website so that it is publicly viewable (read-only) including its live evolution. The live mind map can then be displayed using a large-screen television or monitor at the exhibition stand or made available to participants via a QR code or similar.

## Discussion and conclusions

The ECOUTER method utilising Mind42 has now been implemented and piloted five times (one of which is ongoing):

1. September to November 2014 in collaboration with the Public Population Project in Genomics and Society, Montreal, Canada. *What are the ethical, legal and social issues related to trust and data linkage?* Online, internationally available ECOUTER implementation over a period of several weeks.
2. November 2014 during the ESRC Festival of Social Research, Bristol. *Your medical records - hand over or hands off?* Facilitated digital face-to-face ECOUTER implementation in a public space on one Saturday in a busy shopping centre.
3. June 2015 during the Translation in Healthcare conference, Oxford. *Translation and emerging technologies: what are your views on the social, ethical and legal issues?* Digital face-to-face ECOUTER implementation during the lunch break of an international academic conference.
4. July 2015 during the BioSHaRE tool roll out meeting, Milan. *BioSHaRE Tools - Where to now?* Manual ECOUTER implementation (paper-based, without Mind42) during a day-long workshop.
5. May 2016 to 2017 during the data collection clinic of the Avon Longitudinal Study of Parents and Children (ALSPAC, publicly known as Children of the 90’s) cohort study asking study participants *What areas would you like Children of the 90s to research?* Online ECOUTER over a long time period.

Experience from the above pilots established the efficacy using the free mind mapping platform Mind42 during an ECOUTER. While it was an appropriate solution for the initial specification the pilots did, however, highlight a series of critical limitations and technical issues that need to be resolved before the full potential of the ECOUTER methodology can be realised.

Mind42 is free to use because it generates revenue via targeted advertising, with ECOUTER initiators having no control over the advertisements users are exposed to. Advertising has the potential to distract or influence ECOUTER contributors; users are able to remove adverts only by paying a fee to Mind42. In addition, there are concerns around confidentiality and analysis given that the data sits with Mind42, a company located in Austria. Data are therefore are subject to Austrian law.

Furthermore, the inclusion of several additional features are required within a collaborative mind mapping tool, tailored to the ECOUTER process, to facilitate and strengthen data analysis. These include:

### Enhancements to facilitate ECOUTER management

- Advanced permission management is essential during an ECOUTER to manage users and user groups. This could be used to help define administrator, moderator and contributor roles during an ECOUTER and ensure secure use of the mind map (e.g. preventing contributions from being modified or deleted by others).

### Enhancements to user experience

- Advanced mind map formatting and customisation will enhance readability and user experience. Mind42 has limited formatting capabilities, with only basic methods to format the size of text and colour of mind map branches. As an ECOUTER mind map grows, it can become difficult to navigate the volume of contributions without the use of more advanced formatting features such as bold or italic faces, multiple fonts, font size and text colour. Furthermore, it would be useful for researchers to customise publication grade mind maps for visual impact.
- Agree/disagree buttons would allow contributors to agree/disagree with contributions made by others and to enable researchers to gauge agreement with a comment among the stakeholder community.

### Enhancements for ECOUTER analysis

- Categorisation of contributors would provide researchers with additional information about participants which may be relevant to the ECOUTER question (e.g. level/area of expertise, gender, age) whilst still retaining their anonymity.
- advanced analytics such as activity auditing would assist researchers in understanding and evaluating how the mind mapping tool is used by contributors during an ECOUTER process.

Finally, reliance on third party freeware poses risks to long life-cycle research projects because the software may change substantially in functionality and/or terms and conditions. The software can also shutdown, fail to be maintained or have software errors fixed. An open source self-built solution may be preferable for long term sustainability as an ECOUTER tool and mind mapping service that addresses both researcher and user requirements.
