# Supplementary material for: Digital Methodology to implement the ECOUTER engagement process
Source: F1000Res. 2017 Jan 13;5:1307. Originally published 2016 Jun 9. [Version 2] doi: 10.12688/f1000research.8786.2 (PMC4911626; doi:10.12688/f1000research.8786.2)
Supplement: Peer review history

# Peer review: Referee response for version 2

**DOI:** 10.5256/f1000research.11112.r20243

**Published:** 2017-02-15

**Authors:** Danya F. Vears

**Affiliations:** 1 Department of Public Health and Primary Care, Centre for Biomedical Ethics and Law, Katholieke Universiteit Leuven, Leuven, Belgium

I am satisfied that the reviewers have addressed all the comments made by the reviewers.

I have read this submission. I believe that I have an appropriate level of expertise to confirm that it is of an acceptable scientific standard.

# Peer review: Referee response for version 2

**DOI:** 10.5256/f1000research.11112.r19296

**Published:** 2017-01-17

**Authors:** Michael Morrison

**Affiliations:** 1 HeLEX – Centre for Health, Law & Emerging Technologies, Nuffield Department of Population Health, University of Oxford, Oxford, UK

I am satisfied that the revised version addresses the main points raised in the previous referees comments.

I have read this submission. I believe that I have an appropriate level of expertise to confirm that it is of an acceptable scientific standard.

# Peer review: Referee response for version 1

**DOI:** 10.5256/f1000research.9456.r14267

**Published:** 2016-06-16

**Authors:** Michael Morrison, Teresa Finlay

**Affiliations:** 1 HeLEX – Centre for Health, Law & Emerging Technologies, Nuffield Department of Population Health, University of Oxford, Oxford, UK; 2 Centre for Health, Law and Emerging Technologies, University of Oxford, Oxford, UK

This article describes a novel method for generating responses, opinions and perspectives from stakeholders in a given issue, using a digital engagement processes. The article represents a useful overview of this particular technique and represents a worthwhile contribution to what is an increasingly important area of research. This appraisal comes with the caveat that the article could be improved by addressing the following issues relating to the article content:

 Given that a forthcoming paper describing the ECOUTER process and authored by the same research group is explicitly referenced in the introduction, it would be helpful to have a clearer delineation of how the article in F1000Research is distinct from the forthcoming paper (i.e. what is the *specific* aim of this paper), or why two separate papers describing the ECOUTER process are warranted.

 The authors state that mind-mapping data generated through the ECOUTER process are analysed using ‘social science methods’. This description is unduly vague. As the ECOUTER process generates primarily textual data, it would seem that methods of textual analysis, whether qualitative (e.g. grounded theory-driven thematic analysis) or quantitative (e.g. content analysis) are the most relevant and most applicable subset of social sciences methods (with appropriate methodological additions if visual data is also collected).

 The concept of ‘mind mapping’ is employed but is not explained. It is not necessarily self-evident and should be explained in more depth or a suitable reference provided.

 Implementation online: As pointed out by other reviewers, email addresses can be used to identify individuals especially when combined with other data.

We have read this submission. We believe that we have an appropriate level of expertise to confirm that it is of an acceptable scientific standard.

# Peer review: Referee response for version 1

**DOI:** 10.5256/f1000research.9456.r14352

**Published:** 2016-06-14

**Authors:** Danya F. Vears

**Affiliations:** 1 Department of Public Health and Primary Care, Centre for Biomedical Ethics and Law, Katholieke Universiteit Leuven, Leuven, Belgium

This paper constitutes a valuable contribution to the literature. The title appropriately represents the article and the abstract nicely summarises the content of the paper. All components of the article have been explained to a high standard and are appropriate to the authors' assessment of the mind mapping tool as an appropriate digital methodology for the ECOUTER engagement process. The conclusions drawn are balanced and justified given the assessments made.

 I would encourage the authors to consider elaborating on the following minor points for clarity:

  

1. The paper states that the participants' remain anonymous to both the other participants and also the researchers. However, when implementing ECOUTER online using Mind42, an account is generated for the participants using their email address (which often includes participants' names). Can the administrator see who has registered? Can they see which participants are saying what and if they are part of the research team do they think this has any impact on the study?
2. How can it be assured that the seeded ECOUTER mind map is unbiased and representative of the current state of the literature/debate on the topic?
3. On page 3 (column 2, paragraph 3) the authors state that the facilitator moderates contributions. Could they very briefly clarify what this involves?
4. In the discussion and conclusions, they list 5 successfully implemented pilot studies, the last of which is stated to be running from May to November 2016. Is this year correct? If so, I would exercise caution in calling something that was only set up one month ago and is set to run for another five months "successful".

Well done to the authors on a very interesting initiative and a good assessment of the strengths and limitations of their current approach.

I have read this submission. I believe that I have an appropriate level of expertise to confirm that it is of an acceptable scientific standard.
